# Expressions of “Ikizurasa” in Posts on X (Formerly Twitter) in Japan in 2023: Descriptive Analysis

**DOI:** 10.2196/70613

**Published:** 2025-08-22

**Authors:** Kanami Tsuno, Azusa Arimoto, Yuki Imamatsu, Yumiko Kobayashi, Miho Satoh, Tomoyuki Miyazaki

**Affiliations:** 1 School of Health Innovation, Kanagawa University of Human Services, Yokosuka, Japan; 2 Department of Community Health Nursing, Graduate School of Medicine, Yokohama City University, 3-9 Fukura Kanazawa-ku, Yokohama, 236‐0004, Japan, 81 45-787-2756; 3 Center for Promotion of Research and Industry-Academic Collaboration, Yokohama City University, Yokohama, Japan; 4 Department of Fundamental Nursing, Graduate School of Medicine, Yokohama City University, Yokohama, Japan

**Keywords:** Japan, social media, social networking, suicide, young adult, youth

## Abstract

This study analyzed all public X (formerly Twitter) posts in Japan in 2023 and identified a substantial number mentioning *ikizurasa* (pain of living), with notable fluctuations over time. The findings suggest a link between *ikizurasa* and stress in minority groups, particularly minority ethnic and gender groups.

## Introduction

In Japan, deaths from suicide declined during the early phase of the COVID-19 pandemic but later increased, especially among women and people younger than 40 years [1]. Depression and hopelessness are key predictors of suicidal ideation and behavior [2,3].

Recently, *ikizurasa* (“pain of living”) has drawn attention as a factor in adolescent mental health issues, including *hikikomori* (“being confined,” a term used to describe deep social isolation) and suicide [4]. *Ikizurasa* was defined by the Japanese Neuropsychiatric Society as “impairment and suppression of independent social relationship formation.” It has also been described as involving solitude, anxiety, low self-esteem, hopelessness, and anger [5]. However, quantitative research focusing on *ikizurasa* remains scarce.

Social media behavior has been linked to mental health, particularly among youth. A cross-sectional study in Japan found that posting phrases like “want to die” on X (formerly Twitter) was significantly associated with suicidal thoughts and behaviors [6]. A meta-analysis also reported that frequent social media and smartphone use correlates with suicidal tendencies [7].

This study aimed to examine how often *ikizurasa* was expressed and when these expressions peaked on X in Japan, which has the second-largest X user base after the United States. In 2023, X was used by 65.7% of teenagers, and 81.6% of people in their 20s—much higher rates than among older generations or on other platforms [8]. As X allows anonymous, text-based posting, it offers a space where users may express their emotional distress openly.

## Methods

### Overview

All X public account data from January 1, 2023, through December 31, 2023, were obtained using the official X application programming interface (version 2). The search query was 生きづら (*ikizura*) or 生きにくい (*ikinikui*; both mean “pain of living” or "hard to live"). The analysis was performed in Python.

### Ethical Considerations

This study used only secondary data without identifiable individuals.

## Results

**Figure 1.**
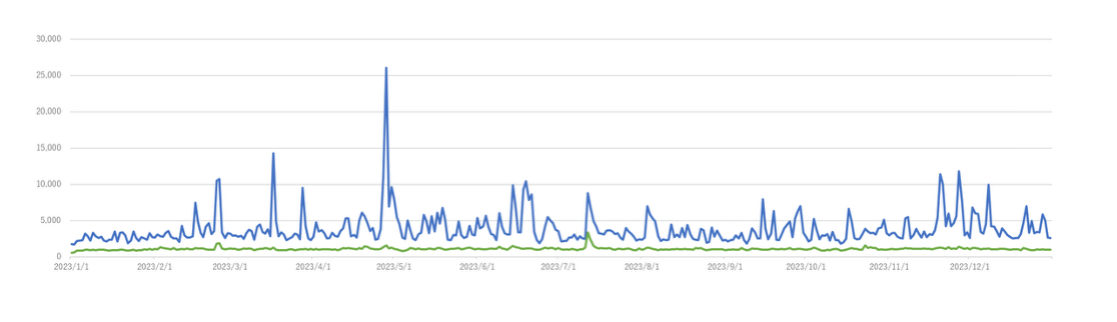
Time series of X posts. The blue line shows the total number of posts (including original posts and reposts), and the green line shows the number of original posts.

In 2023, 1,399,746 posts mentioned *ikizurasa*; 397,517 were organic (ie, excluding reposts or quotes). Figure 1 shows the daily number of posts. The overall average was 3835, with a peak of 26,063 on April 29. Organic posts averaged 1089, peaking at 3414 on July 13, with another peak in late February.

On April 28, an interview article titled “Difficulties in Life as a Minority” [9] was published, reporting on the experiences of discrimination of an ethnically Uyghur member of the House of Representatives, Eri Arfiya, and many X users mentioned the article. On July 12, the news of the death (by presumed suicide) of a well-known transgender celebrity, Ryuchell, was shared with the X community. Many mentioned the news by discussing *ikizurasa* as a transgender person and expressing their shock at Ryuchell’s sudden death that day.

The late February peak was triggered by a post from a US celebrity in Japan, who noted that the lack of English on train tickets may cause foreigners to feel *ikizurasa*.

Table 1 shows the age distribution of the X users who posted using the term *ikizurasa*. Most users who posted *ikizurasa* were in their 20s and 30s.

**Table 1.** Age distribution of X users who posted using the terms *ikizura* or *ikinikui*.

Age (years)	Men, %	Women, %
≤19	1.19	9.66
20‐29	28.57	39.20
30‐39	33.33	32.95
40‐49	22.62	15.34
≥50	10.71	2.84

## Discussion

This study aimed to examine the frequency and timing of posts using the term *ikizurasa* on X in Japan, where the platform is particularly popular among younger generations. Our results showed that over 1.3 million posts containing the term *ikizurasa* were recorded in 2023, with notable peaks in late April and mid-July. These peaks appeared to coincide with news events involving ethnic and gender minorities, suggesting that expressions of *ikizurasa* may be linked to the stress of being part of a minority group.

Previous studies have documented strong associations between minority status—such as ethnicity or gender—and poor mental health outcomes [10,11]. Consistent with this literature, our findings indicate that individuals from minority backgrounds may be more likely to experience and publicly express feelings of *ikizurasa*, reflecting psychological distress tied to discrimination and marginalization.

This study has limitations. It focused only on posts using specific Japanese terms, possibly excluding other expressions of distress. As the analysis relied on public social media data, user authenticity, intent, and demographics could not be verified, limiting interpretation.

Even so, the findings suggest that social media can serve as a real-time indicator of psychological distress, especially among younger and marginalized groups. Integrating such monitoring into public health strategies may help detect emerging mental health issues and support timely, targeted responses.
